# Efficient Training Procedures for Multi-Spectral Demosaicing

**DOI:** 10.3390/s20102850

**Published:** 2020-05-17

**Authors:** Ivana Shopovska, Ljubomir Jovanov, Wilfried Philips

**Affiliations:** TELIN-IPI, Ghent University—IMEC, St-Pietersnieuwstraat 41, B-9000 Gent, Belgium; Ljubomir.Jovanov@UGent.be (L.J.); Wilfried.Philips@Ugent.be (W.P.)

**Keywords:** RGB, NIR, multispectral, demosaicing, deep learning, data sampling, active learning

## Abstract

The simultaneous acquisition of multi-spectral images on a single sensor can be efficiently performed by single shot capture using a mutli-spectral filter array. This paper focused on the demosaicing of color and near-infrared bands and relied on a convolutional neural network (CNN). To train the deep learning model robustly and accurately, it is necessary to provide enough training data, with sufficient variability. We focused on the design of an efficient training procedure by discovering an optimal training dataset. We propose two data selection strategies, motivated by slightly different concepts. The general term that will be used for the proposed models trained using data selection is data selection-based multi-spectral demosaicing (DSMD). The first idea is clustering-based data selection (DSMD-C), with the goal to discover a representative subset with a high variance so as to train a robust model. The second is an adaptive-based data selection (DSMD-A), a self-guided approach that selects new data based on the current model accuracy. We performed a controlled experimental evaluation of the proposed training strategies and the results show that a careful selection of data does benefit the speed and accuracy of training. We are still able to achieve high reconstruction accuracy with a lightweight model.

## 1. Introduction

Multi-spectral imaging systems have a broad range of applications in the area of remote sensing, computer vision, camera-based security systems, etc. Such applications include image enhancement, dehazing, quality inspection, object classification, and the characterization of materials.

The near-infrared band (NIR, 700–1100 nm) is part of the active, or reflected infrared band, with wavelengths closest to the visible light spectrum (400–700 nm). The photometric properties of these two spectral bands are distinct. An image obtained at a specific wavelength represents different information compared to another image captured at another wavelength. While the edges between objects in images captured in the visible and NIR bands match, the intensities and intensity differences are different and depend on the object material. These properties make NIR complementary to the visible light range in numerous applications.

In terms of image acquisition, the similarities between NIR and visible light are that both can be acquired through the same optical path and that silicon-based sensors are sensitive to both bands. Recently, single-sensor cameras for simultaneous acquisition of multi-spectral data have gained popularity in the research community. Using a single optical system and a single sensor for both RGB and NIR modalities alleviates the burden of precise optical calibration and misalignment correction, in contrast to multi-camera, spatial scanning or multi-shot approaches. In our work we will focus on the simultaneous acquisition of RGB and NIR images by a single sensor.

The generalization from RGB to multi-spectral imaging can be achieved by placing an array of different wavelength-selective band pass filters in front of the sensor, denoted as a color filter array (CFA) or a multi-spectral filter array (MSFA). In the case of RGB-NIR imaging, the most practical modification is to replace one of the green pixels of the Bayer pattern [1] by a NIR pixel. In the literature, various configurations of the multi-spectral CFA have been proposed [2] and optimized for different applications. In this paper we will focus on the uniform, Bayer-like pattern.

Regardless of the filter configuration, extending the sensitivity range results in the decreased spatial sampling density of one or more spectral channels. Demosaicing is a crucial step for the recovery of missing information in the imaging pipeline and involves accurate color reconstruction and the alignment of edges. Since each band is sampled at a different spatial location, interpolation artifacts are typically prominent around edges. Looking at wider regions along the edges allows for sophisticated, edge-aware interpolation algorithms [3,4]. In our approach we will rely on a multi-resolution, deep convolutional neural network (CNN) that combines local and spatially broader information.

Due to the complexity of deep neural networks, learning to model accurate data representation requires substantial amounts of training samples. However, using complete images creates a lot of information redundancy in the training samples. For example, natural images contain significant portions of flat regions of low variability. Through uniform random sampling, that proportion is reflected in the training datasets.

Such data redundancy negatively influences the learned representation by introducing bias, a shortcoming that is well known in the image processing literature. Numerous dictionary learning algorithms for sparse representation as well as dimensionality reduction techniques have been focusing on learning compact and informative data representations [5,6]. Another strategy is designing effective instance selection algorithms by means of a specific selection criteria [7].

In this paper we aim to perform data selection for the purpose of training a generative CNN for demosaicing. Some of the principles in this paper are inspired by the tools used in the compressed sensing and dictionary learning literature, thus making a connection between these topics. This focus is on modifying the uniform random sampling scheme to a selective or weighted random sampling so as to achieve a comparable performance and lower training complexity compared to training with a full dataset.

### 1.1. Contributions

**Figure 1:**
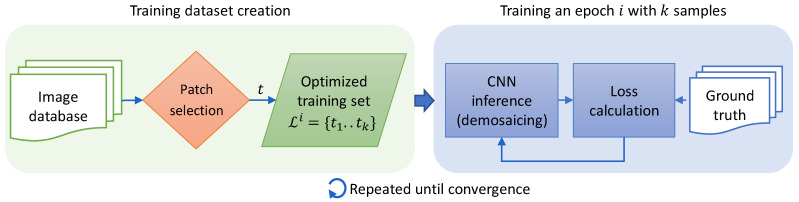
Block diagram of the training pipeline for a single epoch, from an input image database to loss calculation and back-propagation. The proposed data selection techniques apply to the orange diamond shape in the block diagram.

In our prior work [8] we proposed a neural network-based method for demosaicing raw RGB-NIR images using two different sampling patterns. Based on this work, this paper extends the focus towards data sampling and training procedures. More specifically, we propose to carefully select a smaller, informative subset from a large training data set, with the goal to decrease training time and to obtain a more general model. The complete training pipeline is presented with a block diagram in Figure 1, with the orange block representing data selection.

Our first proposed idea is to form clusters in the space of training samples and to train the network only with selected samples that represent all samples in the clusters. With this approach the variability among the selected samples will be high, therefore carrying a lot of information, while ignoring redundant samples. The training data consists of four-channel square patches extracted from a set of training images.

The second approach presented in this paper performs a selection of training samples adaptively, based on the reconstruction error. More specifically, we propose to create a training dataset at each training epoch by statistical sampling and favoring samples from critical regions with a large reconstruction error.

Moreover, we experimented with the objective of demosaicing. Instead of focusing merely on improving the peak signal-to-noise ratio (PSNR) as a standard metric in literature, we simultaneously optimize the structural similarity index (SSIM) as a perceptive quality measure. Some sources in the literature suggest that the combination of metrics yields superior results compared to optimizing each metric alone [9], and our experiments support this finding as well.

In this paper we also decreased the number of network coefficients compared to our prior work [8] by 65% to improve training and run-time speed at the cost of a slight performance decrease. We trained a smaller model by reducing the dimensionality of the convolutional filters. The new, lighter model is comparable in performances to the original one, with 35% of the original number of parameters.

### 1.2. Paper Structure

In Section 2 we present an overview of prior work in the literature, relevant to our research. In Section 3 we define demosaicing as a reconstruction problem and we point out the challenges to solving the problem. In Section 4 we describe the proposed ideas for data selection to improve the training of the CNN from a more theoretical viewpoint, while in Section 5 we elaborate on practical considerations. In Section 6, we describe the modification of the training loss function that we incorporate in this paper compared to our prior work. The experiments and the results are presented in Section 7 and the conclusions from the paper and future work directions are presented in Section 8.

## 2. Related Work

For decades, numerous research efforts have been dedicated to accurate, full-resolution reconstruction from color mosaic images. Many of these works were designed to restore images within the RGB domain. However, the same principles are applicable for the multi-spectral case.

**Figure 2:**
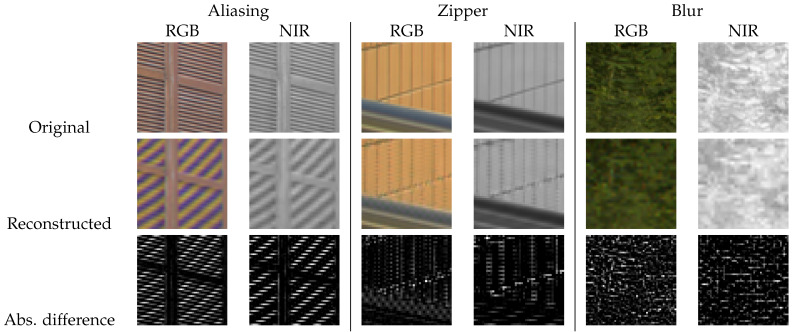
Examples of the most prominent artifacts of demosaicing: aliasing, zipper, and blur.

Image demosaicing is an image interpolation (or up-sampling) problem, and therefore shares many of the challenges present in the problem of single-image super-resolution (SISR) [10]. For example, super-resolution (SR) and demosaicing are ill-posed inverse problems without a unique solution. Moreover, the complexity of the problem and amount of missing details increase with the up-scaling factor. Lastly, assessing the quality of the result can be ambiguous and application dependent. The most noticeable artifacts that occur from image interpolation include aliasing, zipper structures, and blur. Figure 2 shows examples of these artifacts.

Modern super-resolution methods based on deep learning models, such as SRCNN [11] and VDSR [12], show superior performances compared to classical algorithms. These methods perform simple early up-sampling and refine the results using a convolutional neural network. Analogous to this in the demosaicing literature is the early bilinear interpolation applied on mosaic images, before being fed into a neural network to refine the output [13]. Another alternative is to process low resolution inputs and incorporate up-sampling in the cascade of network layers [14,15]. Our proposed method does not involve any initial interpolation and it uses the full-resolution mosaic with zeros at the missing pixel locations.

In a recent work [16], three popular deep learning design concepts have been adapted into a framework for spatial and multi-spectral interpolation from color input mosaics. The architectures include residual network, multiscale network, and parallel-multiscale network. The paper evaluates the effectiveness and computational complexity of the three approaches using synthetic and real data and finds the multiscale properties highly beneficial for the up-sampling task. The parallel-multiscale network achieved the highest reconstruction quality.

In spectral reflectance reconstruction from trichromatic RGB samples, the goal is to reconstruct high-dimensional reflectance vectors from low-dimensional camera responses. Usually this involves both spatial and spectral interpolation, based on a transformation learned from large collections of training samples. The data redundancy problem has been addressed with different sample optimization techniques [17,18,19], with a common requirement of maximizing diversity among the selected samples. In [20], the samples are selected based on local color and texture descriptions of the neighborhood around each pixel, and the results show that including texture improves the quality of the optimized dataset.

To mitigate the negative influence of data redundancy, Birodkar et al. [21] studied three popular classification image datasets and identified 10% of the images as redundant, based on their similarity in a semantic space. In [22], the Active Dataset Subsampling (ADS) approach uses an ensemble of deep neural networks (DNNs) to estimate the uncertainty of each training sample, and discards the samples with the lowest uncertainty. A novel idea for dataset compression was proposed in [23], where the distribution of a large training dataset is "distilled" into a smaller dataset, with a distribution that is an approximate of the original.

Gharbi et al. [14] proposed a convolutional neural network for joint denoising and demosaicing of color images. In this paper, the authors developed an algorithm for detecting challenging samples for demosaicing, according to which only about 40% of all samples in a standard dataset are useful for training. The reported results of training with the reduced, challenging subset are numerically comparable and visually superior to simple random sampling.

The goal of instance selection algorithms [24,25] is to sub-sample a large training set, so that the new, smaller subset is less noisy or redundant and offers high modeling accuracy. Ref. [24] distinguishes two broad categories: Wrapper and filter methods. The approaches in the former group select instances based on the accuracy obtained by a classifier, while the ones in the latter group use an empirical selection criterion which is not necessarily based on the classifier.

However, the problem of over-fitting to difficult examples is commonly observed in the literature and is referred to as the fixation problem in [26]. The “on-demand” approach proposed in [26] attempts to overcome the fixation problem by generating new training instances in targeted difficulty levels, based on the model’s current performance.

Inspired by these conclusions, we explore the idea of sampling difficult samples more densely and adaptively, while at the same time including easy samples in the training set to prevent over-fitting. Unlike most of the methods explained above, we construct a new training dataset before each epoch. Therefore, we are restricted to a simple and fast instance selection technique to avoid a significant increase in the training time. Our goal is to keep the overall training time lower compared to training with a large set without instance selection.

In terms of training objectives, the most popular choices for image restoration problems are minimizing the mean squared error ($L_{2}$ norm) and the mean absolute error ($L_{1}$ norm) between the output and ground-truth. The metric $L_{1}$ is less sensitive to outliers than $L_{2}$ and has recently gained increased interest, especially in the context of denoising. Minimizing the $L_{2}$ norm on the other hand maximizes the PSNR, which is often the main requirement for restoration. For achieving perceptually pleasing results, some researchers rely on a multi-scale structural similarity index (MS-SSIM) [27], with certain approximations to make it differentiable.

The authors of the study conducted in paper [9] experimented with a deep neural network for image reconstruction, optimized with respect to a combination of $L_{1}$ or $L_{2}$ loss with a loss based on the structural similarity index (SSIM) [28]. Their conclusion is that training with a combination of $L_{1}$/$L_{2}$ and a multi-scale SSIM-based loss results in images of higher accuracy to ground-truth and higher subjective visual quality compared to using each metric alone. We adopted this approach in the proposed method and trained the demosaicing network with a combination of $L_{2}$ and two-scale SSIM loss functions.

## 3. Full Resolution Color Image Reconstruction

Reconstructing full-resolution color images from sub-sampled outputs is one of the fundamental processing steps in modern digital cameras. As discussed above, typically a selective color filter array is used to spatially sample different wavelengths. In RGB-NIR imaging, four spectral components are spatially sub-sampled. A reconstruction of the full resolution color components requires interpolating the missing values from the sampled ones.

We will assume a general image observation model that involves a CFA for sampling different light bands [29,30] formulated as:

(1) y=Fx+n

where y is a vector form of the observed sensor data, $x={\left[x_{R},x_{G},x_{B},x_{N}\right]}^{T}$ represents the ideal 4-channel image that we aim to restore, and n is additive noise. The operator F models three successive linear operations in the general image formation pipeline: Wavelength-dependent blur B, spectral cross-talk C, and channel sub-sampling S:

(2) F=S(C⊗I)B.

Each of these three operations, as well as the noise n pose different challenges for the reconstruction of the original ideal signals and have been the focus of numerous research efforts in the past decades.

In this paper we focus on the problem of an accurate reconstruction of full-resolution images $\hat{x}$, given spatially sub-sampled images y, without simultaneously treating the other two problems. The color sampling can be defined using the channel sampling matrix S, which corresponds to the underlying color filter array (CFA) and produces the mosaic y in Equation (3). With this model, we will treat the image as a sum of four separate channels: R, G, B, and NIR.

(3) y=Sx.

The matrix S is a diagonal matrix that stores binary values, indicating the color filter arrangement applied to the pixels. The observation y can be rearranged into a four-channel mosaic image with zeros in the pixel locations where the respective color was not sampled.

Following our previous work [8], we focus on reconstructing an approximation $\hat{x}$ of the ideal, full resolution color image x using a convolutional neural network. The goal of the neural network is to perform the inverse of S, which is a non-linear operation over the mosaic input y. The parameters of the CNN are learned by a loss function defined between the output and ground truth.

In our prior work [8] the demosaicing model was based on the U-Net architecture [31], modified to include additional skip connections forming residual blocks. The U-Net consists of a down-sampling path and an up-sampling path connected with a bridge of layers and skip connections from the down-sampling to the up-sampling layers. Residual learning facilitates coping with vanishing gradients and has resulted in improved performances in numerous applications.

**Figure 3:**
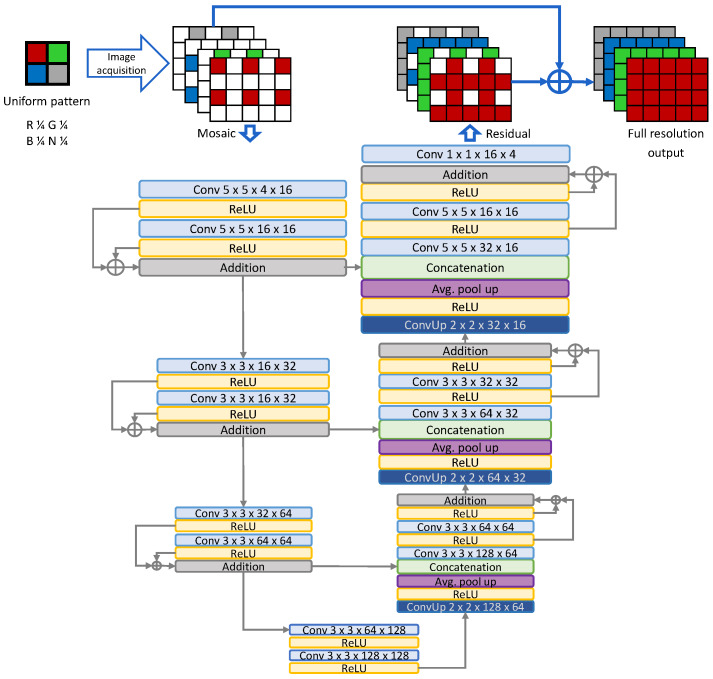
The proposed neural network architecture based on the U-Net model with residual blocks. The network learns to reconstruct the missing information based on the subsampled inputs and combines the output with the original input.

In this paper, we retain the same concept, with small modifications in the architecture. The current architecture is presented in Figure 3, including the size of the filters in the convolutional layers. Compared to prior work, we removed one of the convolutional layers in the first part of the contracting path and decreased the number of feature dimensions in all convolutional layers. The reduction in size is 65%, from 1.45 million trainable parameters to 0.5 million. Moreover, the input to the network are four-channel inputs with sub-sampled channels and zeroes at the missing value locations. The residual learning is extended to keeping the original sampled pixels unchanged and learning to fill-in only the missing values. Ground truth is available in the form of full-resolution RGB-NIR images. Mosaic inputs can be simulated from the ground truth data by channel sub-sampling using the selected CFA pattern.

Training deep neural networks with millions of parameters imposes the need for substantial amounts of data. To achieve generalization, the training data needs to be versatile, which is typically accomplished by acquiring huge training datasets and/or performing data augmentation. However, the fundamental issues with that approach are data redundancy and training time complexity. Our aim is to reduce the training dataset and retain a smaller, highly informative training subset.

## 4. Proposed Data Sampling Strategy

In the domain of demosaicing, the main challenges arise in image regions with strong edges and repetitive textures. Smooth regions do not require sophisticated interpolation methods. For textured regions, neural networks offer stronger modeling advantage over classical methods. Therefore, textured training samples can be considered more informative than smooth ones for discovering the true properties of the data.

In this paper we propose a data sampling scheme in order to improve the training time and/or algorithm performance, as illustrated in Figure 1. On the one hand we take the data variability into consideration to extract maximally informative samples. On the other, we optimize the training set with respect to the current model performance to supply difficult samples. Moreover, in the design of the proposed algorithms we aim to create soft criteria for instance selection to avoid overfitting to a specific sub-problem.

Training with data selected based on a certain criterion has similarities with the principles of active learning [32]. Namely, the hypothesis in active learning is that if the training algorithm is able to choose data from which to learn, it will require less training and achieve better performance.

Let $T=\{t_{j}=\left(x_{j},y_{j}\right),x_{j}\in R^{4},y_{j}\in R^{4},j=1\ldots k\}$ be a large, exhaustive set of training sample pairs $t=(x_{j},y_{j})$ where $x_{j}$ is the ground truth image patch and $y_{j}$ is the corresponding mosaic. Our goal is to create a smaller, optimized training dataset L by selecting samples from T and adding them into L. We propose and analyze two strategies for mining informative samples explained below, and compare them to a baseline which is uniform random sampling.

### 4.1. Uniform Random Sampling

Uniform random sampling is a basic strategy, where the training set $L_{r}$ is created by randomly deciding whether to add each sample *t* in the training set with equal probability. The samples that are included into the training set are the ones with a positive outcome of Bernoulli sampling:

(4) $$L_{r}=\left\{t|z_{t}∼\mathrm{Bernoulli}\left(p\right)=1\right\}$$

where $z_{t}$ is the binary {0,1} Bernoulli outcome for patch *t*, and *p* is the probability for $z_{t}$ to be 1. The probability *p* is fixed for all samples and determines the sampling density i.e., the proportion of samples added from T into $L_{r}$.

Uniform random sub-sampling of data is a fast technique that results in a subset that carries the same amount of information as the original data. It enables representing the original data distribution with fewer samples. However, by sampling uniformly regardless of the locations from the image manifold where data is more difficult to model, the subset may contain many redundant samples that do not contribute towards finding the optimal solution.

### 4.2. Sample Clustering

Our first proposed strategy creates an optimal training dataset $L_{c}$ by clustering the samples based on their low-level features. We consider this approach as passive learning since the current state of the model is not considered during instance sampling. However, drawing from the ideas in active learning, we propose to form a set of highly variable training samples that will cause significant changes in the model during training and lead to faster convergence. To avoid overfitting, a new $L_{c}^{i}$ is generated before each training epoch *i*.

Initially, a large pool of samples $S^{i}$ is obtained by uniform sampling from T (Equation (4)), since working with the whole T is computationally prohibitive for this approach. The samples from $S^{i}$ are then clustered and only the resulting cluster centers are included into the final training set $L_{c}^{i}$.

The sample patches are described by low level features including spectral intensity and texture. For clustering, we rely on the k-means method [33], which is a simple and powerful iterative algorithm for unsupervised data partitioning. The objective of k-means is to minimize the within-cluster variance (WCV), which is equivalent to maximizing the between-cluster variance (BCV), a result of the fact that the total variance of the data set remains constant [34]. Our goal is to obtain a dataset of samples $\left\{t_{1}^{*},\ldots t_{k}^{*}\right\}$ corresponding to the cluster centers $\left\{\mu_{1},\ldots \mu_{k}\right\}$ that minimize the WCV and maximize the BCV:

(5) $$L_{c}^{i}=\underset{\left\{t_{1},\ldots t_{k}\right\}}{\arg\min}\sum_{j=1}^{k}\sum_{\tilde{f_{t}}\in C_{j}}\left|\right|\tilde{f_{t}}-\mu_{j}{\left|\right|}^{2}$$

where $f_{t}$ is the normalized feature vector calculated from ground truth patch *x* in sample $t\in S^{i}$, *k* is the number of clusters $C_{1}\ldots C_{k}$, and $\mu_{j}$ is the mean of the samples belonging to cluster $C_{j}$.

To confirm the hypothesis that the same amount of centroid samples carry more information than uniform random samples, we calculated the average per-feature entropy, based on the normalized features $\tilde{f}$. The average of 100 different random realizations of each type of dataset is presented in Table 1.

It is not surprising that the data sets generated by uniform random sampling have the same entropy, since in both cases the distribution of image patches is sampled in an identical manner. The clusters, on the other hand, are formed such that the variance between the them is high, and therefore they carry more information.

### 4.3. Adaptive Selection Based on Past Error

The second data selection strategy we propose is closer to the idea of active learning. Before each training epoch *i*, a new training set $L_{a}^{i}$ is formed by evaluating the samples in T with the current model (trained up to epoch i−1) and sampling with higher probability the patches that result in a higher reconstruction error. This strategy gives the model the opportunity to adapt by learning from examples that are more difficult to describe, based on its own modeling ability.

Analogous to this approach in active learning is entropy-based uncertainty sampling for classification problems. The fundamental difference is that we do not make predictions of the future model outcomes, since ground truth is available, and we can calculate the past error as a criterion for mining new samples.

Creating the training dataset $L_{a}^{i}$ based on the current model θ can be formulated as selecting only samples *t* that follow the rule:

(6) $$L_{a}^{i}=\left\{t=\left(x,y\right)|z_{t}∼\mathrm{Bernoulli}\left({\tilde{l}}_{\theta }\left(\hat{x},y\right)\right)=1\right\}$$

where ${\tilde{l}}_{\theta }\left(\hat{x},y\right)$ is an error metric normalized in the range [0,1], defined as difference between the reconstructed patch $\hat{x}$ and the corresponding ground truth *y*. The random variable $z_{t}$ introduces randomness in the decision whether the sample *t* will be included in $L_{a}^{i}$. Samples with a larger error will have a higher chance of being included in the next round of training, to change the model faster and towards more optimal solution. Sampling with variable probability based on fitness allows generating a set of difficult samples, as well as including some smaller proportion of samples that are easy to model so as to prevent overfitting.

## 5. Practical Implementation

In this section we will explain the practical aspects of the implementation of the proposed methods with respect to computational complexity and dataset size. In our experiments we rely on the RGB-NIR Scene Dataset [35] and generate the training sets by cropping patches of size 64×64×4 from the images. The dataset contains 477 pairs of matching RGB and NIR images with an average size of 700×1000 pixels.

### 5.1. Data Clustering

For the sample clustering approach, the patches are described by low-level features of intensity and texture. The feature vectors consist of average intensities per channel in the patch (4 values), and the histogram of oriented gradients (HOG) obtained by a weighted gradient angle contributions by the corresponding magnitudes (5 values). The intensity information is important to accurately model the spectral properties of the data. Including samples with different patterns and edge orientations is beneficial for learning to perform more accurate interpolation around edges.

Based on these features, a bisecting k-means strategy is carried out, recursively splitting the data into two sub-clusters, as long as the intra-cluster variance is larger than a variance threshold, or the number of samples in the cluster exceeds a cluster size threshold. Finally, the selected training samples are the ones closest to the centroids of the clusters in the feature space.

**Figure 4:**
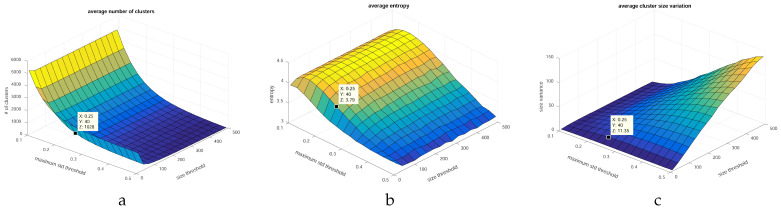
Grid-search for optimal stopping criteria with respect to different cluster properties: (**a**) average number of clusters, (**b**) average entropy, (**c**) cluster size variation. The selected point is a tradeoff between processing time, variability and cluster balance.

Setting a threshold on the cluster variance serves to control the data compression strength. The cluster size limit is imposed to prevent data imbalance. We have experimentally chosen the stopping thresholds. In Figure 4 we present a coarse grid-search of parameters, carried out simultaneously over viable ranges of values, analyzing three different properties of the output datasets.

Figure 4a shows the average number of clusters resulting from the variance and size threshold combinations. Choosing a too-small number of clusters may result in high intra-cluster variance and severe under-representation of interesting samples. Having too many clusters, however, will decrease the compression rate and may not sufficiently suppress redundancy in the final set. The average entropy per feature in the output set (Figure 4b) is related to inter-cluster variance, which we aim to maximize. The third property (Figure 4c) is the variation in the sizes of the resulting clusters (in terms of cluster cardinality). This value is an indicator of cluster imbalance.

Based on these three properties we can make a tradeoff between processing time (number of clusters) and quality of the dataset (entropy and size balance). Since there is no global optimum, we chose a value around the knee point of the curvature in Figure 4a. The selected threshold pair should not greatly decrease the entropy from the highest possible value in that range (Figure 4b). Finally, the sizes of the clusters should be balanced, which is ensured by analyzing Figure 4c.

The motivation for top-down hierarchical k-means clustering is three-fold. First, the number of clusters does not need to be specified in advance, it can be controlled by constraining the cluster variance and cardinality.

**Figure 5:**
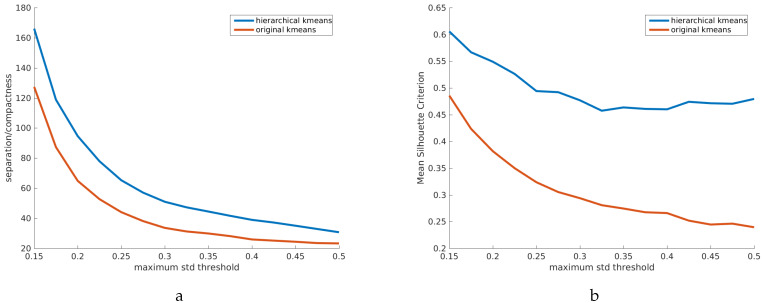
Clustering performance metrics of original vs. hierarchical k-means for a range of cluster variance thresholds. We have compared: (**a**) the ratio separation/compactness and (**b**) the mean Silhouette criterion, which show that hierarchical k-means provides more separated, compact and consistent clusters.

Moreover, the quality of the clusters obtained by hierarchical clustering tends to be higher than that of the original k-means (Figure 5). To compare the two strategies, we evaluated the quality of the resulting clusters, based on two common criteria: The ratio of separation/compactness (Figure 5a), and the Silhouette criterion (Figure 5b), both of which we aim to maximize. Large separation and small compactness values correspond to well-defined clusters. High Silhouette criterion is an indicator of high cluster consistency.

In this experiment, the cluster variance, as an input parameter for hierarchical k-means, was gradually increased, resulting in fewer, larger, and more diverse clusters. For the original k-means, the number of clusters in each step was set to match the hierarchical approach. To make a fair comparison, we have not constrained the cluster size.

The third motivation for hierarchical clustering is the time complexity. The complexity of the original k-means is $O(n^{2})$, while that of the hierarchical k-means is lower, $O(n\log_{2}\left(n\right))$. In Table 2 we present the breakdown of the number of operations required in the original vs. hierarchical k-means.

### 5.2. Adaptive Training

In the second proposed sampling strategy, we adaptively selected the samples in $L_{a}^{i}$ by evaluating the reconstruction quality at all pixel locations in the training images. The training patches were randomly cropped from the training images, with the probability proportional to the average reconstruction error ${\tilde{l}}_{\theta }\left(\hat{x},y\right)$ around each pixel, in a window of size equal to the patch size.

For practical reasons, we can equivalently re-formulate the sampling rule defined with Equation (6):

(7) $$L_{a}^{i}=\left\{t=\left(x,y\right)|{\tilde{l}}_{\theta }\left(\hat{x},y\right)>z_{t};z_{t}∼U\left(0,1\right)\right]\}$$

where $z_{t}$ is a uniform random variable in the range [0,1]. The samples included in the next training epoch $t^{*}=\left(x,y\right)$ are pairs of ground truth patches *x*, corresponding sub-sampled mosaics *y* at pixel locations where the condition in Equation (7) are met. This process is carried out for each training image separately, to increase the variability among the selected samples and to prevent overfitting to the same globally difficult cases.

The reconstruction error ${\tilde{l}}_{\theta }$ can be measured by the mean squared error between the reconstructed and the ground truth training images. We will evaluate several different choices for the error metric in the experiments section.

**Figure 6:**
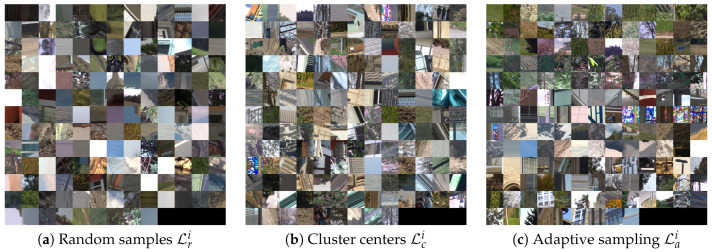
Examples of RGB patches from each type of dataset. Random sampling results in many smooth patches, while clustering and adaptive sampling select more textured patches for more informed training.

For illustration, Figure 6 shows examples of color patches which belong to training datasets created with each of the data selection methods explained above. In the dataset of uniformly selected samples (Figure 6a) there is a significant portion of flat patches. The samples generated by clustering, based on color and texture features, is shown in Figure 6b. Here the samples are more variable and include various textures and edge orientations. On the third example we present a set of patches selected based on their reconstruction loss. This example shows that the difficult cases are mainly textured regions.

## 6. Training Objectives

In many image restoration problems, including super-resolution and demosaicing, the peak signal-to-noise ratio (PSNR) is one of the standard metrics for evaluating image quality. PSNR is calculated based on the mean squared error (MSE) between the reconstructed output image $I_{out}$ and a ground-truth, reference image $I_{ref}$, for images in the range of [0–255]:

(8) $$PSNR=10\log_{10}\frac{255^{2}}{MSE(I_{out},I_{ref})}.$$

As a metric for image quality, PSNR is not well correlated with the perceived reconstruction quality. On the other hand, the structural similarity index (SSIM) is a perceptually inspired metric for the structural correspondence between images. Typically, SSIM is calculated based on small windows at the same location in two images. The similarity between two windows $w_{x}$ and $w_{y}$ at pixel position *u* is:

(9) $$SSIM\left(u\right)=\frac{2\mu_{x}\mu_{y}+C_{1}}{\mu_{x}^{2}+\mu_{y}^{2}+C_{1}}\frac{2\sigma_{xy}+C_{2}}{\sigma_{x}^{2}+\sigma_{y}^{2}+C_{2}}$$

where $\mu_{x}$ and $\mu_{y}$ are the mean values in the corresponding windows, $\sigma_{x}$ and $\sigma_{y}$ are the variances in each window, and $\sigma_{xy}$ is the covariance. Constants $C_{1}$ and $C_{2}$ are small numbers used for normalization, and in our method they are set according to standard practice, to 0.001 and 0.009, respectively.

Among the most popular training loss functions in image reconstruction literature are the MSE ($L_{2}$ norm), Cityblock distance ($L_{1}$ norm), and SSIM-based loss. The metric $L_{1}$ is less sensitive to outliers than $L_{2}$. Minimizing the $L_{2}$ norm maximizes PSNR, however it is less correlated to perceived difference between two images. SSIM helps in preserving the structure, however, depending on the window size used to calculate its terms, it can cause artifacts either around the edges or in smooth regions [9].

The MSE loss for a reconstructed patch $\hat{x}$ compared to a reference patch *y* of size *N* is the average squared difference between the intensities from all pixel locations *u*:

(10) $$l_{MSE}=\frac{1}{N}\sum_{u=1}^{N}{\left(\hat{x}\left(u\right)-y\left(u\right)\right)}^{2}.$$

The Cityblock (or $L_{1}$) distance, used as loss function, is defined as the average absolute difference between the intensities from all pixel locations *u* in two compared patches $\hat{x}$ and *y*:

(11) $$l_{L_{1}}=\frac{1}{N}\sum_{u=1}^{N}∥\hat{x}\left(u\right)-y\left(u\right))∥.$$

Since SSIM can be implemented as a differentiable function with some approximation, it can be used as a loss function for training. Similarly to the approach in paper [9], our method calculates multi-scale SSIM (MS-SSIM) on two different scales, approximated by Gaussian windows with different standard deviations, $\sigma_{1}$ and $\sigma_{2}$. The multi-scale, SSIM-based loss function can be defined as:

(12) $$l_{MS-SSIM}=1-\frac{1}{N}\sum_{u=1}^{N}\left[\lambda_{1}SSIM_{\sigma_{1}}\left(\hat{x}\left(u\right),y\left(u\right)\right)+\left(1-\lambda_{1}\right)SSIM_{\sigma_{2}}\left(\hat{x}\left(u\right),y\left(u\right)\right)\right].$$

The conclusions of the analysis of loss functions for image restoration with neural networks in paper [9] is that due to the convergence properties, surprisingly, the $L_{1}$ loss can outperform $L_{2}$ according to various metrics, including PSNR. Additionally, a model trained with a combination of $L_{1}$ and MS-SSIM loss, outperformed the models trained with each individual loss functions.

In a similar fashion, in the proposed method we carried out small-scale experiments with training using $L_{1}$, $L_{2}$, and MS-SSIM individually, or using a combination of any of the $L_{1}$ or $L_{2}$ norms with MS-SSIM. For example, the combined loss of MSE and SSIM-based error is:

(13) $$l=l_{MSE/L_{1}}+\alpha l_{MS-SSIM}.$$

Combining $L_{1}$ with MS-SSIM is performed in the same fashion. In the current experiments we set $\lambda_{1}=0.5$, and α=10 to balance the error ranges. The Gaussian window standard deviations were determined experimentally and set to $\sigma_{1}=1$ and $\sigma_{2}=3$, for a window of size 7×7 pixels.

## 7. Experiments and Results

For all experiments, we relied on the RGB-NIR Scene Dataset [35] to train and compare different models. Approximately 75% of the images in the dataset were reserved for training, and the remaining 25% for validation purposes. For a final evaluation and comparison with the literature we relied on three additional, public datasets of RGB and NIR images: The Freiburg Forest Dataset [36], IVRG [37], and OMSIV [38].

To investigate and evaluate the influence of each proposed data sampling strategy, as well as the training objective functions, four different, controlled experiments were carried out: (1) Evaluation of the optimal distance metric for clustering-based instance selection (Section 4.2); (2) evaluation of the optimal error metric for adaptive sampling, (Section 4.3); (3) evaluation of the optimal loss function for training the network for demosaicing (Section 6); and (4) comparative evaluation of the proposed strategies. Finally, a model according to the best performing approach will be trained.

For a controlled study, we attempted to make the experimental conditions equal, by minimizing differences in initialization and hyper-parameters. Therefore, the parameters of the neural network (convolution filters and biases) were randomly initialized and stored, with the goal of being re-used as an initial hypothesis for all experiments. Moreover, the number of training samples per epoch was fixed to 2500 patches with a size of 64×64×4. The models were optimized using the Adam optimizer [39], with a learning rate of $10^{-3}$, weight decay of $10^{-3}$, and batch size of 25. The comparative experiments were carried out for 50 epochs and stopped early, since we are predominantly interested in the trend of the learning curves. The final model will be fully trained until convergence.

The experiments performed in this study are small-scale, due to the limited number of images in the dataset. Training with more samples from the same set of images may add redundancy, without any significant added information. Moreover, the difference between the random samples and the optimized samples will become smaller, making the comparison less conclusive. Nevertheless, we expect that the conclusions from the small-scale experiments can be extrapolated for large-scale datasets and intend to investigate this in the future.

### 7.1. Optimal Distance Metric for Clustering-Based Instance Selection

As explained in Section 4.2, the training dataset is obtained by clustering a large pool of randomly selected patches from the training images. The initial set of random patches $S^{i}$ contains 50,000 samples. By hierarchically applying k-means, the samples are clustered into two subsets, and recursively continue to be divided until any of the stopping criteria is met. In this experiment, the cluster size was limited to 50, and the variance threshold adjusted so that the number of clusters was approximately 2500. Out of them, exactly 2500 were selected for a controlled comparison. For each distance metric tested, a different variance threshold was selected. The centers of the clusters form $L_{c}^{i}$.

Different datasets $L_{c}$ were created using four different distance metrics in the clustering algorithm: MSE, cityblock distance, cosine distance, and correlation-based distance. The motivation to include cosine and correlation-based distance metrics in the evaluation was inspired by the spectral reconstruction techniques [17,18,19].

**Figure 7:**
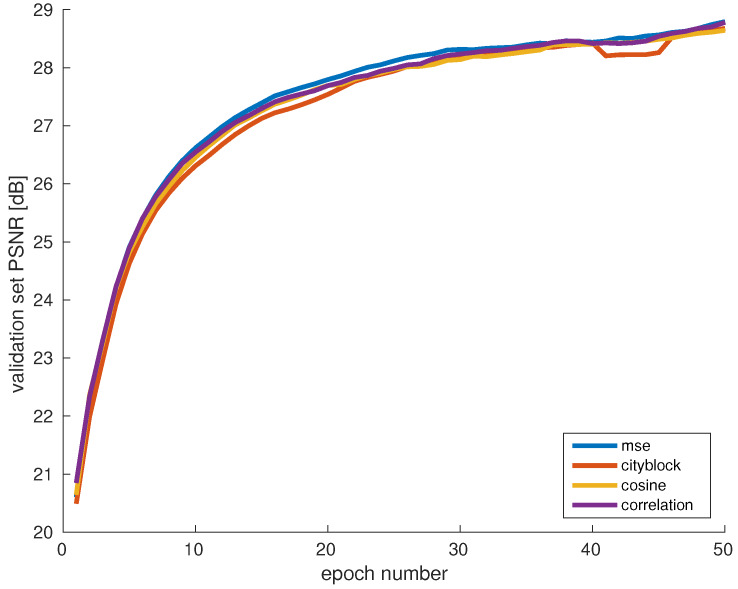
Evaluation of the effect of different distance metrics for data clustering. The experiments show no significant impact of the choice of distance metric on training accuracy.

In Figure 7, the learning curves of the models trained with each dataset are compared. The y-axis denotes the average PSNR between the ground truth patches and the reconstructed patches of the validation set. The results of this experiment indicate that in the current framework, the choice of distance metric does not have a noteworthy influence on the training performance. Nevertheless, the mean squared error performs slightly better than the other distance metrics in the earlier training stages.

We suspect that the similarity in the results comes from the fact that the training process does not critically depend on the actual cluster properties, if the selected center samples are sufficiently diverse. Another reason could be that drawing samples from a limited number of training images results in overlap between the datasets. Furthermore, there is a close connection between the cosine and correlation distances with the Euclidean ($L_{2}$) distance. Both cosine and correlation distances perform normalization based on the assumption that the vectors lie in the Euclidean space. Finally, by dividing the clusters based on their within-cluster variance, the algorithm tends to be biased towards the $L_{2}$ distance.

### 7.2. Optimal Error Metric for Adaptive Instance Selection

For the second instance selection strategy, we investigated the impact of the error metric on the training performance. The probability of sampling new training patches from various locations in the training images is proportional to the reconstruction error in those locations. For each pixel, the average error is calculated based on its local neighborhood (Section 4.3).

The motivation to compare different error metrics comes from the variability in metrics used as evaluation criteria in the demosaicing literature [9,20]. Additionally, for multi-spectral reconstruction, paper [18] implies that the differences between multi-spectral signals can be measured more effectively with the Chebyshev distance.

In this experiment we evaluated five different error metrics: MSE, SSIM, Chebyshev distance, cityblock distance, and PSNR. Since PSNR and SSIM describe the similarity between two inputs, the probability for the selection of new samples is with inverse proportion.

**Figure 8:**
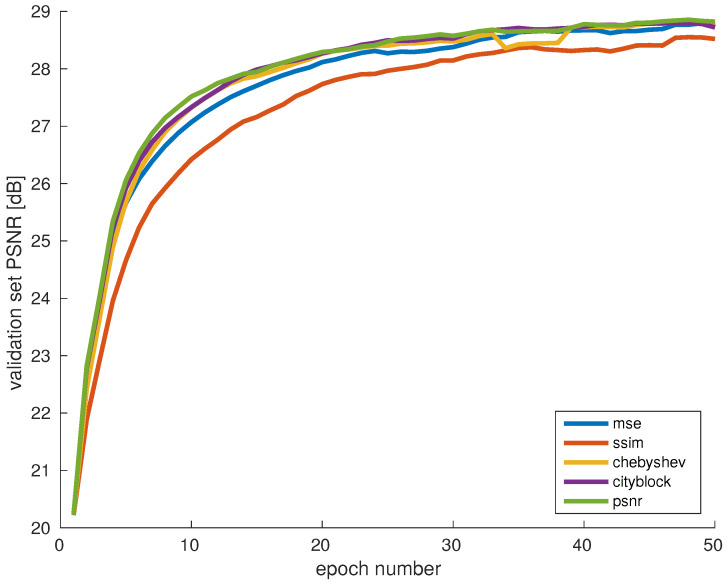
Evaluation of different error metrics for adaptive data selection. The numerical error metrics do not significantly affect the quality of the generated dataset. SSIM as a perceptual metric pefrorms slightly worse, due to the differences in the definition of image quality between SSIM and PSNR.

Figure 8 shows the learning curves for each of the error metrics that were investigated. Interestingly, SSIM scores lower than the other metrics on this graph, while the other metrics tend to be more similar. The main differences are noticeable in the earlier training stages, while the learning curves reach the same level in the later phase. PSNR performs slightly better than the other metrics, which is not surprising since performance is also measured with PSNR. Furthermore, the small difference between MSE and PSNR could be a result of the normalization with respect to the peak value in the calculation of PSNR.

On the other hand, the lower scores of SSIM indicate that focusing only on examples with textures and structures that are difficult to reconstruct is not the optimal strategy for training. The reason may be in the difference between metrics such as MSE or PSNR that estimate absolute errors and whole SSIM is designed to measure perceptual similarity and describes perceived changes in structural information.

### 7.3. Optimal Loss Function for Training the Demosaicing Network

In the previous two experiments, the training loss function was the standard MSE. With the third experiment, the goal is to investigate the effectiveness of other loss functions as well, motivated by the conclusions in [9]. The loss functions evaluated include MSE, $L_{1}$, MS-SSIM, and the combinations of MSE with MS-SSIM and $L_{1}$ with MS-SSIM (Section 6). For this experiment we reverted to the uniform subsampling for computational efficiency.

**Figure 9:**
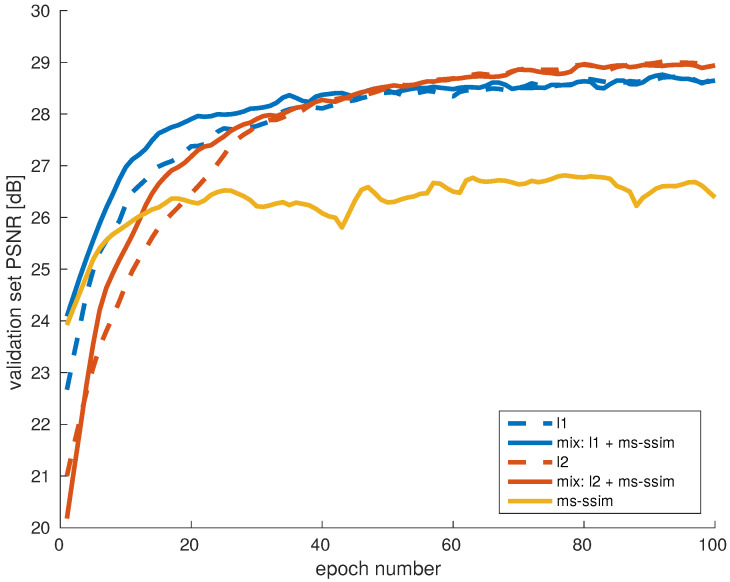
Evaluation of different loss functions for training a demosaicing network. A combined loss improves training speed, compared to minimizing each metric individually. MS-SSIM is sensitive to image details and can converge to a subpoptimal solution with respect to PSNR.

In Figure 9, we present the learning curves of training with different loss functions, evaluated according to PSNR on the validation set. In this case, 100 training epochs per model were completed, so that the convergence using different losses can be observed on a longer scale. Interestingly, in the beginning the model trained using $L_{1}$ (blue, dashed curve) reached higher PSNR faster than the one using $L_{2}$ (red, dashed curve). After the first 50 epochs, the model trained using $L_{2}$ continued to improve and converged to a more optimal solution.

The model trained using MS-SSIM corresponds to the green curve in Figure 9. It converged earlier and to a suboptimal solution. From the visual inspection of the results, one explanation could be that the colors in the reconstructed images appear washed-out, resulting in a low PSNR. Since SSIM is designed to focus on image structure and is not very sensitive to color shifts in flat areas, this is not a surprising result.

The combinations of $L_{1}$ and $L_{2}$ with MS-SSIM are represented with the full lines in the corresponding colors. As Figure 9 shows, the combined loss noticeably improved the training speed, compared to minimizing each metric individually. This experiment also shows that SSIM complements well the standard loss metrics for the problem of demosaicing.

From the conclusions of paper [9] and from our analysis, we suppose this may be due to several reasons. For example, MSE leads to accurate color reconstruction, however it may get stuck in a local minimum. On the other hand, due to its multi-scale nature, MS-SSIM helps in a more accurate reconstruction around edges.

**Figure 10:**
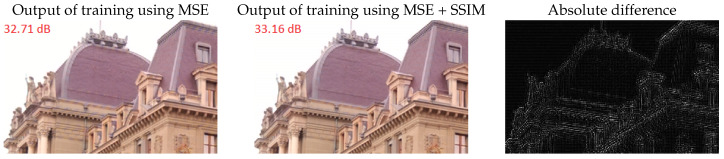
Effects of training with different loss functions (**left**: mean squared error (MSE), **middle**: combination of MSE and structural similarity (SSIM) loss), mostly prominent around object edges (**right**).

Figure 10 shows an example obtained with models trained with the two types of loss functions. As shown, the main differences between the two outputs were found around the edges in the image. Visually, there were small noticeable differences along the thin, repetitive lines along the base, and the top of the roofs, where aliasing was reduced by the mixed loss.

### 7.4. Comparative Evaluation of the Proposed Strategies

As a final comparative experiment, we compare the proposed strategies against each other, and against a baseline which is uniform sampling. Apart from the training dataset, all other aspects of the training are identical.

**Figure 11:**
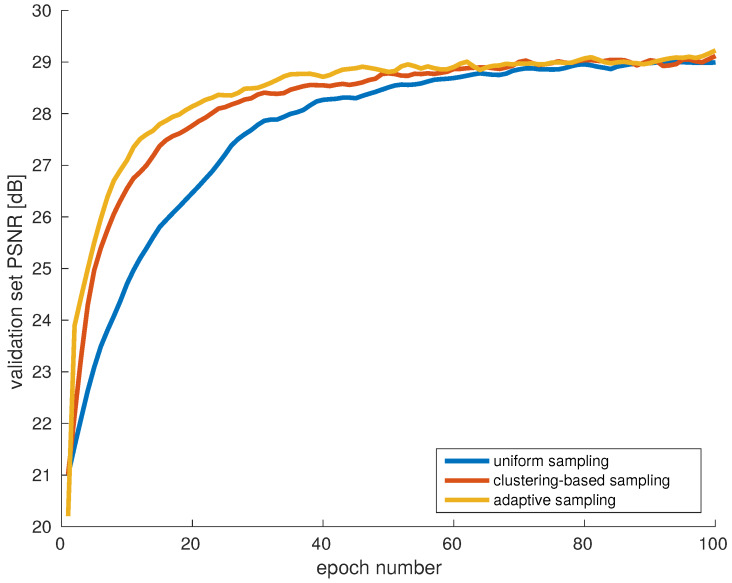
Evaluation of the two proposed strategies (clustering-based and adaptive sampling) against a uniform random sampling baseline. The proposed strategies clearly improve the training efficiency.

In Figure 11 we present a comparison of the learning curves during 100 epochs, obtained with the best performing variant of each data selection procedure. For time efficiency, MSE alone was used as a loss function. The reconstruction quality was measured by PSNR on the validation set. The steeper the learning curve, the faster the network learned, and the higher the curve, the better the accuracy of the model.

Compared to uniform sampling, the proposed strategies clearly produced more optimal datasets with which the model could efficiently be trained. In the early stages, the difference in PSNR was considerable, which shows the effectivenes of a carefully optimized dataset.

Even in the intermediate training stages, the proposed approaches showed better performance than the baseline. For example, to reach PSNR of 29 dB, it took 100 epochs for the random selection model, 80 for the clustering based approach, and 62 for the model trained by adaptive sampling.

The curves became close in the later traning stages, which can be explained by the limited dataset availability in the current setup. Due to randomness in choosing samples in each epoch, after many iterations the model would encounter many different examples from the training images, increasing the overlap between the different datasets.

### 7.5. Dataset Generation Complexity

The main conclusion from the previous experiments is that a careful selection of informative training samples does positively influence the required number of training iterations and accuracy.

Another valuable evaluation is the time complexity for generating the datasets. The goal was to estimate how scalable the proposed ideas were with the growing availability of the dataset. Since we focused on improving the training speed, the dataset creation process should not significantly add to the total training time.

**Figure 12:**
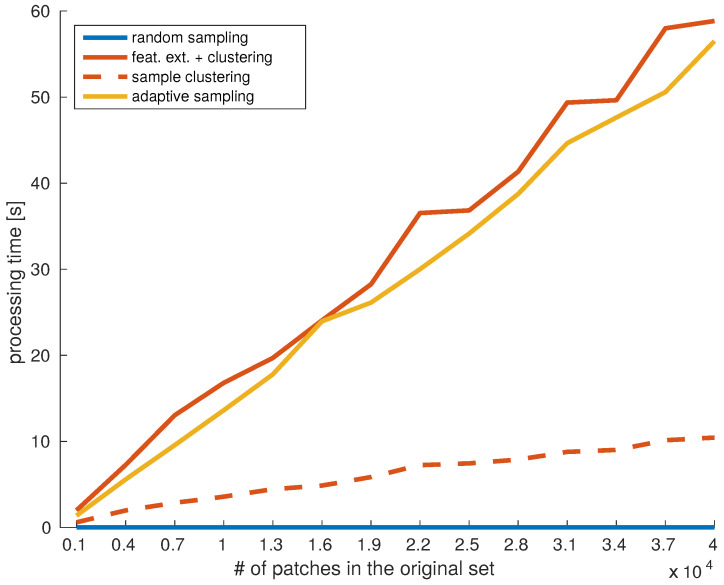
Time required to process and subsample an increasing number of candidate patches, by each data sampling strategy.

In Figure 12 we compared the time required to create each type of dataset, with the increasing dataset size. In each test, the pool of potential candidate patches was linearly increased, and the target number of samples was set to 5% of that pool. The goal was to evaluate the processing time of the proposed approaches, with respect to increasing data availability. The presented results averaged from 3 repetitions of the same experiment.

The absolute running times in this experiment depend on several factors, including the optimality of the implementation and the processing hardware. For example, in the clustering-based approach, with our current implementation, around 78% of the processing time was spent on feature extraction, and the rest on clustering. On the other hand, the patch evaluation in the adaptive sampling was carried out on the GPU, in batches of 1000 patches.

Therefore, we are more interested in the slopes of the lines presented in Figure 12. As expected, the fastest approach was random sampling (blue line) since the only operation was cropping from random locations. Its running time linearly depended on the number of images and lied in the order of $\left[10^{-3},10^{-4}\right]$ seconds. The time required for clustering-based data selection increased significantly faster with the increase of data (red lines). To get a more complete analysis, we separately provided the time required only to cluster the data (dashed line), and the total processing time of the approach, including feature calculation (full line). With a faster feature extraction method, the approach could become time efficient. The adaptive sampling (orange line) required demosaicing all patches in the pool before deciding which ones to sample, which is also a time-consuming operation.

Nevertheless, the total processing time of one epoch should be considered when choosing a data selection approach. In some cases, it may be more beneficial to train with a larger, randomly sampled dataset rather than to dedicate the same amount of time for pre-processing. On the other hand, a well-defined training set would result in faster convergence and fewer training epochs.

### 7.6. Demosaicing Performance

Finally, using each of the two proposed approaches, a different model was trained until convergence. The hyperparameters were tuned for each model individually, to reach best performance. Based on the conclusions from the experiments explained above, the final strategies for the proposed approaches are the following:

1. Hierarchical k-means clustering is applied to a large pool of potential samples. $L_{2}$ is used as a distance metric to form clusters in Euclidean space. The loss function that is minimized is the combined loss of MSE and MS-SSIM. We will denote the model that corresponds to this strategy as data selection-based multi-spectral demosaicing using clusteing (DSMD-C).
2. Adaptive data sampling is carried out based on the MSE error metric for consistency with the training loss and due to the negligible difference with the other possibilities explored in the experiment. The loss function is also the combined loss of MSE and MS-SSIM. This model will be denoted as data selection-based multi-spectral demosaicing using adaptive sampling (DSMD-A).

To evaluate the performances objectively, we compared the reconstruction quality with existing demosaicing methods for RGB and NIR images from the literature. The selection of algorithms for comparison was limited to methods with publicly available code that used the uniform pattern, so as to ensure the experiment could be controlled. However, we expect that with powerful deep learning algorithms, the reliance on the pattern layout will not be strong [40], if the patterns have the same spectral sampling density.

In Table 3 we present the PSNR and SSIM scores calculated between the reconstructed images and their corresponding ground truth images. We carried out tests on three publicly available datasets: Freiburg Forest Dataset [36], IVRG [37], and OMSIV [38]. In the table, the methods in the first group are classical methods from the literature with publicly available codes. The method denoted as RGB-NIR-Unet is the model proposed in our prior work [8]. As indicated, this model is larger in number of trainable parameters compared to the models proposed in this paper. In our prior work, the training strategy differs from the current one in terms of dataset size per epoch and the hyperparameters. Therefore, the training performance per number of epochs is not directly comparable.

The best performances of the two approaches proposed in this paper are listed in the last two rows of Table 3. With only 35% of the total number of parameters compared to [8] (a decrease of 65% of the number of weights and biases), the new models achieved a similar reconstruction accuracy. Moreover, they clearly outperformed existing classical state-of-the-art methods.

Freiburg Forest Dataset contains 136 images with a size of 480×860, with natural scenes and fewer strong, high-frequency details due to the scene type. This makes the images easier to reconstruct, which is reflected in the high PSNR values that algorithms achieved on this dataset. IVRG contains 25 test images (512×768) with more variable and textured content, including images of charts often used for testing performance limits. Therefore, IVRG is more challenging for demosaicing algorithms. OMSIV contains 533 images of size 256×256, and the difficulty of this dataset comes from poorer image quality and the presence of noise.

**Figure 13:**
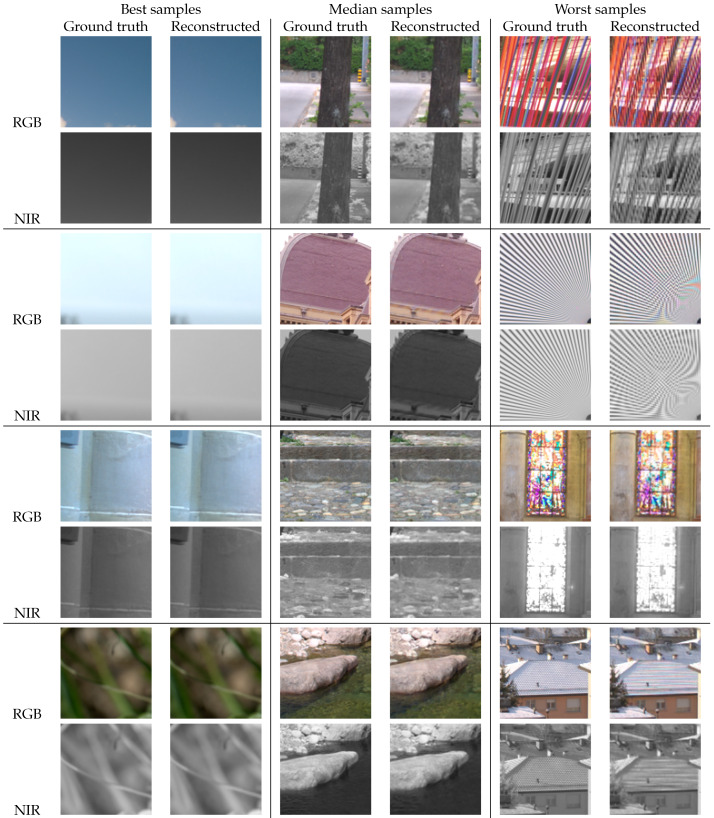
Selected crops from test images with the best, median, and worst performance.

For visual inspection, in Figure 13 we have selected representative regions from test images in the IVRG dataset, and the reconstruction results with our DSMD-A model. We show examples of some of the best samples, some examples with typical, median quality, and some of the ones with the lowest PSNR. Smooth regions without strong, saturated colors are the easiest to reconstruct and they comprise the set of best reconstructed samples. The examples with median quality consist of regions with typical textures and edges. In these examples too, there are no noticeable reconstruction artifacts. The main challenges remain to be fine, repetitive textures. However, this is one of the fundamental limitations for all demosaicing methods. Examples such as the ones that were selected are less common in typical datasets. Still, even in those regions, the distracting artifacts are reduced in comparison with other methods, which is confirmed by the PSNR and SSIM values in Table 3.

## 8. Conclusions and Future Work

Deep learning models are complex, with enormous number of trainable parameters. To train them robustly it is necessary to provide sufficient training data, with a lot of variability. This makes deep learning highly time-consuming, and the information redundancy can have adverse effects on training. Therefore, strategies for instance selection and data reduction are gaining increasing interest for numerous machine learning problems. In this paper we investigated different approaches for optimal dataset subsampling to train a deep learning model for multispectral demosaicing. Two general strategies were considered.

The first proposed strategy optimized the training subset by maximizing the variability among the selected samples. To obtain a variable and representative training set, a hierarchical data clustering was adopted. The set of cluster centers obtained by k-means was a subset of high variance. Therefore, it maximized the magnitude of the model change with each parameter update and led to faster and more optimal convergence. Another benefit was that with large datasets, this technique could be carried out off-line and with pre-defined features based on domain knowledge.

For efficiency and to find more optimal clusters, we performed hierarchical clustering, with k-means (k = 2) applied in each branch of the space-partitioning tree. Based on our experiments, we found this technique not overly sensitive to the distance metric used in clustering in terms of final dataset quality.

The second proposed strategy was adaptive sampling and focused on self-guided training, where the samples were selected based on their reconstruction error. The model could choose the most interesting samples and improve the data representation faster and more precisely.

In the experiments we evaluated different error metrics to measure the difference between the reconstructed and reference images, based on which samples were selected. The learning curves that were compared indicate that for optimizing PSNR, using SSIM as an error metric was not optimal. This may be due to the design of SSIM as a measure perceptual similarity and therefore not being consistent with metrics such as PSNR. Another explanation is that focusing entirely on the reconstruction quality of high-frequency details can lead to overfitting.

In a controlled experiment, we compared the two proposed techniques against a baseline which is uniform random sampling. The results showed that both methods performed well, with adaptive sampling reaching a higher PSNR faster than the clustering-based approach.

Additionally, motivated by the findings in the literature, we tested several loss functions for training. The experiments indicated that combining the mean squared error with a loss function based on the structural similarity index benefited the training even further.

Finally, we trained a model based on the conclusions from the previous experiments and chose the best performing variants. Specifically, the new model was trained using the adaptive sampling strategy, and with a loss function which is a combination of MSE and MS-SSIM. Compared to our prior work, we achieved a comparable reconstruction accuracy on three public datasets, with a model reduced in size for 65%, retaining only 35% of the total number of parameters.

In future work, we will focus on real multispectral data that will provide interesting opportunities to further investigate the proposed concepts. A more comprehensive solution in the image reconstruction pipeline will consider the spectral sensitivity of the sensor to each wavelength, as well as the spectral correlation between channels. Furthermore, we will experiment with combining the strengths of the proposed approaches, depending on the size of the available training image datasets. An interesting research direction could be to form partitions in a large data space and based on the reconstruction error of representative samples, to sample more densely in the more difficult partitions. Finally, we will focus on larger datasets and apply the findings in this paper to other deep learning-based computer vision problems.

## Figures and Tables

**Table 1:** Average per-feature entropy in a training data set.

Dataset	Number of Samples	Average Entropy
Large set S, uniform sampling	10,000	3.38
Small set $L_{r}$, uniform sampling	1000	3.36
Small set $L_{c}$, cluster centers	1000	4.20

**Table 2:** Time complexity breakdown for k-means vs. hierarchical k-means data clustering.

	Original k-Means	Hierarchical k-Means
Distance calculation	k×n×d	
Finding minimum	k×n	
Worst-case	k=n	k=2
1 iteration	$n^{2}\left(d+1\right)$	2n(d+1)
Number of iterations	*M*	$M\times \log_{2}\left(n\right)$
Total	$M\times n^{2}\left(d+1\right)$	$M\times \log_{2}\left(n\right)\times 2n\left(d+1\right)$

*n*—samples; *d*—dimensions; *k*—clusters; *M*—max. iterations.

**Table 3:** Comparison of the performance of the proposed method with existing classical demosaicing methods from the literature.

Model	PSNR [dB]/SSIM		
	Freiburg [36]	IVRG [37]	OMSIV [38]
Bilinear	41.19/0.9740	30.42/0.8999	30.57/0.9067
Binary-tree edgde sensing demosaicing (BTES) [41]	40.94/0.9714	30.42/0.8968	29.23/0.8805
Least-square, multispectral demosaicing (LMSD) [42]	40.48/0.9654	31.42/0.8944	29.27/0.8656
Multisp. adaptive residual interpolation (MS-ARI) [3]	41.85/0.9787	33.01/0.9419	31.23/0.9261
Monno (uniform pattern [43])	41.12/0.9688	32.39/0.9308	30.34/0.9007
RGB-NIR-Unet [8] (1.45 M param., prior work)	44.20/0.9850	34.87/0.9531	33.52/0.9424
Proposed DSMD-C (clusters, L2 + SSIM, 0.5 M param.)	43.27/0.9813	34.31/0.9474	33.24/0.9397
Proposed DSMD-A (adaptive, L2 + SSIM, 0.5 M param.)	44.21/0.9850	34.72/0.9516	33.49/0.9438
